# Impact of hepatitis B virus and hepatitis C virus infection on sperm parameters of infertile men

**DOI:** 10.18502/ijrm.v17i8.4820

**Published:** 2019-09-03

**Authors:** Sana karamolahi, Reza Salman Yazdi, Mehrangiz Zangeneh, Mahin Jamshidi Makiani, Behnam farhoodi, Mohammad Ali Sedighi Gilani

**Affiliations:** ^1^Tehran Medical Sciences, Branch Islamic Azad University Tehran Iran.; ^2^Department of Andrology, Reproductive Biomedicine Research Center, Royan Institute for Reproductive Biomedicine, ACECR Tehran Iran.; ^3^Department of Infectious Diseases, Faculty of Medicine, Tehran Medical Sciences, Islamic Azad University Tehran Iran.; ^4^Department of Infectious Diseases, Reproductive Biomedicine Research Center, Royan Institute for Reproductive Biomedicine, ACECR Tehran Iran.; ^5^Antimicrobial Resistance Research Center, Institute of Immunology & Infectious Diseases, Iran University of Medical Sciences Tehran Iran.

**Keywords:** Hepatitis B virus, Hepatitis C virus, Male, Infertility, Sperm

## Abstract

**Background:**

Viral hepatitis is one of the health problems which have the effects on the health issues. It seems that hepatitis B virus (HBV) and hepatitis C virus (HCV) infection have negative impacts on the semen quality and male infertility rate.

**Objective:**

In this study, we aimed to evaluate the effects of HBV and HCV on sperm quality among Iranian infertile men referred to Royan Institute Reproductive Biomedicine Research Center between 2003 and 2014.

**Materials and Methods:**

This retrospective case-control study included 112 HBV positive infertile men and 47 HCV positive infertile men as case group and 112 HBV negative and HCV negative matched infertile men as a control group. All semen analysis and viral parameters assessment was performed in the central laboratory with the same method and instruments.

**Results:**

Sperm count among infertile men with HBV and HCV infection was significantly lower than control group [the mean of the total sperm count 100.95░±░118.59, 118.22░±░141.18, 166.27░±░151.25 (p░<░0.001)]. Sperm motility was significantly decreased in HBV and HCV positive men in comparison to the control group [30.97░±░25.88, 31.09░±░28.72, 40.87░±░23.37, respectively (p░<░0.007)]. The percentage of normal sperm morphology was significantly higher in control group in comparison to HBV and HCV infected group [the mean of the normal semen morphology 3.23░±░3.27, 3.70░±░3.83, 4.51░±░3.15 p░<░0.015]. Although there is a significant decline in liquefaction time in the case group but the viscosity, semen volume, and PH of semen samples were similar in the both case and control groups.

**Conclusion:**

Our results suggest that HBV and HCV infection are associated with poor sperm quality.

## 1 Introduction

Viral hepatitis is one of the major global public health concerns. According to international reports, the prevalence of hepatitis B virus (HBV) in Iran is moderate (1, 2). Although they are hepatic viruses, these viruses have also been found in extrahepatic tissues such as kidneys, ovaries, and testis in the semen. Viral cirrhosis or hepatocellular carcinoma and even death had been reported as pathological complications among patients with chronic viral hepatitis (3). Infertility is a common issue in the world and according to the World Health Organization (WHO) reports, there are around 80 million infertile couples around the world (4). Overall infertility rate and its specific rate among 21–26 years old Iranian male population is 10–15% and 17.2%, respectively (4, 5). The relationship between HBV and hepatitis C virus (HCV) infection and male infertility has been a considerable interest, as the impact of HBV and HCV on the sperm parameters have been studied by many researchers. Lorusso and co-workers reported that sperm concentration, morphology, and viability have been damaged among HBV seropositive infertile men (6). Other studies in this filed showed that some of sperm parameters were impaired in infertile men with chronic HBV infection, and even there was a negative correlation between HBV viral load and sperm parameters (7, 8). Antisperm antibodies were detected among 12% of men who were assessed for infertility in Egyptain patients with HCV infection (9), and in the most of viral hepatitis patients, and antisperm antibodies had been bound to their spermatozoa (10). Hepatic viruses have a negative impact on the passing of the sperm through the female genitalia and reaching to fertilizing space (11). In some cases, exposing sperms with HBV can induce oxidative stress in the cells and may lead to apoptosis. However, there are few studies about the impact of HBV and HCV infection on sperm parameters of infertile men in the national infertility clinics in Iran.

The present study has been performed for evaluation of HBV and HCV impacts on the sperm parameters of infertile men referred to main infertility clinic for Iranian infertile men. We aimed to perform this study in Iran to know if the effect of HBV and HCV infection on semen quality in our area is the same as other countries. If HBV and HCV infection have the effect on the semen quality, we need do more studies about the effects of HBV and HCV infection treatment on the sperm quality improvement.

## 2 Material and Methods

### 2.1 Study subjects

This retrospective case-control study was performed on 157 male patients according to the inclusion criteria: 1) 112 positive patients for hepatitis B surface antigen who confirmed by HBV DNA assay by PCR method and 2) 47 positive patients for HCV antibody, all HCV Ab-positive cases confirmed by HCV RNA assay by PCR method too, and 3) 112 seronegative men for HBV and HCV were selected as a control group. All three groups were referred to the Royan Institute Reproductive Biomedicince Research Center between March 2003 and March 2014 for infertility treatment.

Exclusion criteria was disorders such as hydrocele, varicocele, epididymitis, human immunodeficiency virus (HIV) positive and herpes simplex virus positive infections that have an impact on the sperm parameters. Infertility in this study was defined as a couple which had not pregnancy with experience of one year sexual contact without contraceptive. Semen samples were obtained of infertile men after 3–5 days sexual abstinence.

Serological test was performed by Enzyme-linked immunosorbent assay (ELISA) method. All patients in the case and control group were tested for anti-HCV-Ab, HBs-Ab, HBs-Ag, HBe-Ag, HBe-Ab, HDV Ab, HBc-Ab, HIV-Ab, and *Treponema pallidum* by ELISA kits from Pishtaz-Teb Inc-ex Tehran, Iran company. All HCV Ab and HBsAg positive cases were confirmed by PCR method. All of the laboratory tests were performed in institute central laboratory with the same method and instruments. Sperm analysis data, HBV and HCV serology results and participants’ demographic data were gathered into the study checklist.

### 2.2 Assessment of sperm parameters between two groups

According to study data checklist, semen samples were obtained by masturbation after 3–5 days of sexual abstinence. All samples were allowed to liquefy at room temperature for 30░min prior to analysis. Sperm analysis was assessed based on the last WHO report (12) for variables; including sperm count, volume, PH, viscosity, viability, agglutination, amorphic pattern and abnormal sperm head (double, giant, pin, and round head), tail (double, coiled, short), motility}a) rapid progressive, b) slow progressive, c) non-progressive and d) no motility and sperm morphology. The results were compared between case and control groups.

### 2.3 Ethical consideration

This study protocol was confirmed by the Ethical Committee of the Royan Institute Reproductive Biomedicince Research Center. The research protocol obtained the ethical guidelines of the (1975) Helsinki Declaration and the relevant local regulations. Because of the study was retrospective, we could not take consent from patients.

### 2.4 Statistical analysis

Statistical analysis was performed using the SPSS statistical software for Windows (Statistical Package for the Social Sciences, version 21.0, SPSS, Inc., Chicago, IL, USA). Quantitative and qualitative variables were presented with mean/standard deviation and frequency/percentage, respectively. We assessed the distribution pattern of study variables; statistical analysis for normal variables, independent sample *t*-test and in variables without normal distribution, Spearman's Rho correlation was used for study analysis. We used chi-square for the determination of significant association between study qualitative variables. Two-tailed p░<░0.05 was considered statistically significant.

## 3 Results

### 3.1 Baseline characteristics

Baseline demographic and semen parameters properties of the HBV and HCV positive and negative men in the case and control groups was shown in table I. The semen parameters of 159 seropositive infertile men (112 HBs Ag and 47 HCV Ab positive) with 112 seronegative men for HBV and HCV as a control group were analyzed. Mean age of the participants with HBV and HCV infections and control group were 36.67░±░7.52, 35.54░±░9.51, and 34.40░±░7.8░yr old, respectively.

### 3.2 Sperm count

Although all of infertile men in our study had normal sperm count (15–200 million/ml), the mean sperm count was significantly decreased in HBV-positive (34.75░±░34.63 vs 56.21░±░40.32; p░<░0.001) and HCV-positive (40.33░±░39.40 vs 56.21░±░40.32; p░<░0.001) subjects. According to Spearman correlation analysis, mean of total sperm count had a significant association with HCV infection (rho = -0.038).

### 3.3 Sperm motility

There is a weakly significant correlation between total sperm motility and HBV infection (p░<░0.05), (rho < 0.025). Mean sperm motility subclasses A, AB, and ABC were significantly decreased in the HBV positive in comparison to HBV negative men. In sperm motility subclasses C and D, there was no significant difference between the case and control groups. Sperm motility both totally and in subclasses B, AB, and ABC was significantly decreased in the HCV positive men in comparison to the control group. HCV infection had no significant correlation with sperm motility subclasses A, C, and D (sperm motility: Rapid Progressive; Class A; Progressive, Class B; Total Progressive, Class A░+░B; Non-progressive, Class C; Non-motile, Class D; and Live Ratio, Class A░+░B + C).

### 3.4 Sperm morphology

The percentage of normal sperm morphology was significantly higher in control group in comparison to HBV-infected group (72 (60.49%) vs 47 (39.51%); p░<░0.001). Spearmen rho correlation analysis showed that there is a significant association between sperm morphology and HBV infection (rho < 0.25; p░<░0.01). The frequency of normal sperm morphology in HCV infection was lower in comparison to the control group (p░<░0.05). The sperm liquefaction time in HBV and HCV infection group was significantly decreased compared to the control group. The viscosity, volume, and PH of sperm samples had no significant association with HCV and HBV infection (Table I).

## 4 Discussion

Nowadays, the interest has been focused on the relationship between HBV and HCV infection and male semen quality. In this study, we found that HBV and HCV infected men had decreased semen total sperm count, progressive sperm motility, and impaired morphology in comparison to the matched control group. Although the mean of sperm count in the both, case and control group were within the normal range but total count in HBV and HCV infection was lower than control group (p░≤░0.001). The results of this study showed that HBV and HCV infection have negative impacts on sperm parameters. This study is similar to the previous studies that reported the effects of HBV and HCV infections on sperm parameters. Garolla and co-workers in their study reported that viral infections including HBV and HCV, Adeno-associated virus, cytomegalovirus, papilloma virus, and HIV have a negative impact on fertility rate of men; in addition, these viruses may transfer to their wife or infant (13). Vicari and co-worker reported that although some of the sperm parameters including total sperm count, density, progression, morphology and lifetime were changed in HCV infected patients, HCV infection was not related to infertility rate (8). Lorusso and co-workers in their study on 132 infertile men with HCV, HBV, and HIV infection reported decreased sperm parameters such as sperm concentration, mean of sperm motility, sperm lifetime and morphology in the infected group in comparison to non-infected group (6); in our study too, mean of sperm motility has decreased. Moretti and co-workers observed although sperm quality of infertile men with HBV and HCV infection had not changed, sperm apoptosis and necrosis increased in infected patients (7). Huret and co-worker performed their study on 10 acute hepatitis B and five chronic hepatitis B cases and reported necrospermia and teratospermia in 62% and 46% of patients, respectively, and asthenospermia, low sperm volume and sperm count in 36%, 33% and 27% of patients, respectively (14). Durazo and co-worker assessed semen parameters and serum level of fertility hormones among patients with HCV infection and reported, patients with HCV infection have lower semen parameters and antiviral therapy can improve sperm parameters (15). Garrido in his study on 125 patients with viral hepatitis and 125 matched patients as a control group reported a negative correlation between sperm progression and serum level of CD4+ and positive correlation between sperm motility and hepatitis improvement (16). Garrido and co-workers reported the role of two years’ semen washing as a part of ICSI (Intracytoplasmic Sperm Injection), on HCV and HIV infection in the infertility rate among infertile couples. He reported, there is no significant difference between the infertility rate and quality of semen and infant rate between patients with HBV and HCV infection and other participants. Fertilization rate among patients with HCV infection was lower than patients with HIV infection (16). Ofney and co-workers studied on semen parameters of 57 men with HCV infection. In his study semen parameters such as volume, count and motility was decreased among HCV positive patients. And also, the time of HCV infection had a negative impact on semen volume and motility (17). Zhou XP and co-workers evaluated semen parameters of 916 men (457 HBV positive and 459 HBV negative) who were referred for fertility assistance. In this study, HBV infected men showed lower semen volume, lower total sperm count as well as poor sperm motility and morphology (p░<░0.05) in comparison to the control group as our study. Also, the clinical pregnancy rate was lower among HBV positive patients in comparison to HBV negative patients after ICSI and embryo transfer (p░<░0.05) (18). Fu-Hsiung Su and co-workers evaluated the risk of male infertility among patients with HBV infection based on a national population-based cohort study (19). Their data showed an increased incidence and risk of infertility among men with HBV infection compared with men without HBV infection. In our study, 159 HBV and HCV infected patients showed lower sperm counts, motility and changing morphology. Our data confirm the result of previous studies that mentioned HBV and HCV infection have effects on sperm quality of infertile men.

## 5 Conclusion

In the present study, HBV and HCV infection have significant and negative impact on the sperm parameters. Future studies should be done to assess the role of semen washing protocol in lowering infertility index among patients with HBV and HCV infection, and also, we need to do further studies to assess the effect of HBV and HCV infection treatment on infertility rate.

**Table I:** Result of semen parameters in case and control groups

Semen parameters	Case group with HBV infection (n░=░112)	Case group with HCV infection (n░=░47)	Control group (n░=░112)	P-value
Frequency of normal sperm counts^a^	67 (59.82%)	20 (42.55%)	84 (75%)	< 0.001*
Frequency of normal sperm motility^a^	43 (38.39%)	19 (40.43%)	69 (61.61%)	0.001*
Frequency of normal sperm morphology^a^	53 (47.32%)	19 (40.42%)	69 (67.20%)	0.001*
Total sperm count^b^	100.95░±░118.59	118.22░±░141.18	166.27░±░151.25	0.001*
Total sperm motility^b^	30.97░±░25.88	31.09░±░28.72	40.87░±░23.37	0.007*
Normal sperm morphology^b^	3.23░±░3.27	3.70░±░3.83	4.51░±░3.15	0.015*
Semen volume (ml)^b^	2.97░±░1.53	2.64░±░1.32	3.16░±░1.71	0.16
Semen pH^b^	7.77░±░0.29	7.65░±░0.94	7.79░±░0.09	0.17
Semen liquefaction time^b^	24.79░±░7.95	24.57░±░8.33	22.05░±░5.83	0.01*

* Statistically significant (P░<░0.05)

a. data presented as n (%) b. data presented as mean±SD Note: HCV: Hepatitis C Virus; HBV: Hepatitis B Virus; Human Immunodeficiency virus: HIV; Herpes simplex virus: HSV; Enzyme-Linked immunosorbent assay: ELISA Sperm count with Spearman correlation analysis; Spearmen rho correlation analysis for morphology and motility; Independent student *t*-test was used for statistical analysis

## Conflict of interest

The authors declare no financial interests.
